# Supplementary material for: Epidemiology of multimorbidity in low-income countries of sub-Saharan Africa: Findings from four population cohorts
Source: PLOS Glob Public Health. 2023 Dec 6;3(12):e0002677. doi: 10.1371/journal.pgph.0002677 (PMC10699623; doi:10.1371/journal.pgph.0002677)
Supplement: Attachment — Submitted filename: Response to reviewers.docx [file pgph.0002677.s002.docx]

Dear Editors,

We would like to thank you and the reviewers for taking the time to provide feedback on our manuscript. We found the reviewer comments constructive and helpful. In view of these comments, we have made extensive changes to the manuscript. We believe that these changes have made the manuscript much stronger. Please find our point-by-point response to reviewer comments below.

Yours Sincerely,

Bhautesh D Jani (on behalf of all co-authors)

**Reviewer #1**

The authors describe the epidemiology of multimorbidity in three low income countries in sub-Saharan Africa. The manuscript is generally well written, but I have some minor suggestions to assist in improving readability and clarity.

1. There are several typographical errors including stray upper case letters, inappropriate punctuation and a repeated phrase on page 9 (definition of asthma and epilepsy) that need to be addressed.

**Author response**: Thank you for your comments, this has been corrected.

Changes to Manuscript>Page9>Lines 12-15>Methods: We defined asthma and epilepsy based on as a prior clinical diagnosis or current treatment for the condition. Prior diagnosis of epilepsy (based on medical records) was available only for The Gambia data, while prior diagnosis of and asthma (from self-report at interview,) was available only for the Malawi data.

2. Please clarify whether the blood samples taken from participants in The Gambia were fasting samples and if they were analysed for this study or if results were extracted from the database.

**Author response**: We have clarified that the bloods samples collected from participants in The Gambia were fasting samples.

Changes to Manuscript>Page8>Line 3>Methods: Clinical parameters (weight, height, body composition, blood pressure) were taken and fasting blood and urine samples were collected. ~~and blood and urine samples were collected~~.

3. Please clarify whether blood samples were taken in the Uganda cohort and if so, how they were handled and analysed.

**Author response**: Thank you for your suggestion, this has been clarified.

Changes to Manuscript>Page8>Lines 22-23>Methods: Non-fasting blood samples were taken from the participants in the Uganda cohort.

4. Similarly, please clarify how all blood samples whose results are included in the definition of multimorbidity were analysed.

**Author response**: For each individual condition considered for the definition of multimorbidity, role of blood results have been clearly stated.

5. Please clarify why a previous diagnosis of hypertension was not used as a criterion for defining hypertension.

**Author response**: This has been clarified, previous diagnosis of hypertension (based on self-report or clinical notes.

Changes to Manuscript>Page9>Lines 2-6>Methods: For these analyses, we defined hypertension as a systolic blood pressure measurement of at least 140mmHg, a diastolic measurement of at least 90 mmHg(23) or self-reported use of current hypertension medication or from clinical notes.

6. Please clarify whether HIV was included in the definition of multimorbidity. It is not included in the list on page 9, but is included subsequently.

**Author response**: Thank you for pointing out this error, this has been corrected.

Changes to Manuscript>Page9>Lines 20-21>Methods: Multimorbidity was defined as the presence of two or more of the following conditions: hypertension, diabetes, HIV, obesity, asthma, epilepsy or high cholesterol.

7. Please justify why pregnant women were only excluded from the anthropometric analyses and not from the study as a whole or a sensitivity analysis performed excluding pregnant women. Pregnancy can affect parameters such as blood glucose and blood pressure.

**Author response**: Pregnant women were excluded from estimates of obesity but data on health conditions including epilepsy, asthma, cholesterol, HIV, hypertension, were included in the multimorbidity estimates as these health conditions require management irrespective of pregnancy status and some conditions are either unrelated to pregnancy or may persist beyond pregnancy. A sensitivity analysis excluding pregnant women will not be useful as pregnant women represent less than 1% of all multimorbidity cases included in these analyses.

Changes to Manuscript>Page 9> Lines18-22>Methods: Pregnant women were excluded from estimates of obesity but data on health conditions including epilepsy, asthma, cholesterol, HIV, hypertension, were included in the multimorbidity estimates as these health conditions require management irrespective of pregnancy status and some conditions are either unrelated to pregnancy or may persist beyond pregnancy.

Changes to Manuscript>Page 15>Lines 1-17>Results: Of note, N=41 women were pregnant at the time of cohort recruitment and excluded from the anthropometric measurements for obesity.

Changes to Manuscript>Table 3:>Legend: Excluding pregnant women N=241

8. The authors use the phrase "materially greater" several times in the manuscript-please clarify how this was determined.

**Author response**: The word “materially” was used to emphasise evidence of statistically significant difference in estimates. This has been simplified to “greater” in all instances in the manuscript.

9. The authors assert that females were at higher risk of multimorbidity in all study populations-this was not however the case in The Gambia.

**Author response**: This error has now been corrected.

Changes to Manuscript>Page 23>Lines 6-8 >>Discussion: Furthermore, females were at higher risk of multimorbidity compared to males in all study populations, apart from The Gambia cohort, as observed in previous studies from high- and middle-income countries (27–29).

10. Citation 40 is out of sequence in the discussion. Please ensure that use of upper case letters is standardised in the reference titles.

**Author response**: Thank you for spotting this error, this sequence in referencing has been corrected and changed to 37. Also, all referencing has been amended to reflect the journal style.

11. I suggest citing numbers to 1 decimal place in text and tables.

**Author response**: Thank you for your useful suggestions. We have amended all tables and text to 1 decimal place, apart from presenting incidence rate ratios (where we have limited it to 2 decimal places) and the corresponding confidence intervals and where prevalence estimates where less than 1%.

12. i suggest switching around the row and column variables in Tables 1 and 2.

**Author response**: Tables 1 and 2 have been changed to reflect this.

13. Please use the term "High cholesterol" in Table 4, rather than just "Cholesterol".

**Author response**: Thank you for the recommendation. Table 4 has been amended accordingly. .

15. In Table 5, I suggest explaining that estimates are incidence rate ratios in a table footnote rather than including this in a row.

**Author response**: Table 5 has been amended to reflect the reviewers suggestion.

16. In Table 5, I suggest placing the p values in appropriately placed columns rather than rows.

**Author response**: We found that adding p-values in columns was making table 5 too wide, with too many columns and struggling to fit the table in the page width (with landscape format).

**Reviewer #2**:

A relevant and timely paper on the epidemiology of multimorbidity in sub-Saharan African countries that addresses research gaps.

Minor

1. Please add line numbers to your revised manuscript and refer to these when highlighting corrections in your response to reviewers

**Author response**: Thank you for this useful suggestion; line numbers have been added to the manuscript pages.

2. page 6 methods paragraph 1: add country name in the initial intro to the datasets (e.g. "Kiang West Longitudinal

Population Study (The Gambia)...")

**Author response**: Country names have been added to this sentence.

Changes to manuscript>Page6>Lines 1-4: We used cross-sectional data from: the Malawi Epidemiology and Intervention Research rural and urban cohorts, 2013-2017 (https://datacompass.lshtm.ac.uk/961/); the Kiang West Longitudinal Population Study (The Gambia); and the General Population Cohort in Kyamulibwa (Uganda).

3. page 12 Table 1: standard for sociodemographic indicators to be in left-hand column, and country data to be in subsequent columns (i.e. table rotated -90degrees) - same for other tables in document which are in this format. Column "female number" would better read as "female respondents"

**Author response**: These changes have been implemented for tables 1 and 2, thanks.

4. page 15 table 3: combine rows with number of cases and crude prevalence into one (XX (XX%)) for easier reading

**Author response**: Table 3 has been amended as suggested.

Moderate

5. We tend to overestimate the impact of smoking and alcohol consumption on morbidity in non-western contexts as this relationship is well established in caucasian populations in high income countries. However there are very different genetic and cultural drivers of morbidity and I would have liked to see this better highlighted: for instance whilst the authors do mention level of active lifestyles and diet in their discussion as possible additional risk factors, it is not clear whether such information was collected and whether it could be used to better inform the models in table 5. This is underlined by the data from Malawi in particular - the lowest levels of alcohol and smoking of the three included countries, but had a trend of the highest prevalences of morbidity. The authors are probably already aware of this introductory synopsis to the Hebe Gouda study (and the Gouda study itself):

https://www.thelancet.com/journals/langlo/article/PIIS2214-109X(19)30370-5/fulltext

which also supports the relevance of risk factors such as diet, air pollution, and exercise over alcohol and smoking.

If no such data is available, It would be great to see an expanded discussion of the likely additional risk factors that are SSA or LMIC specific.

**Author response**: Thank you for your insightful comments. Unfortunately, such data are not available for these analyses, and we agree that this is an important limitation, which we have acknowledged.

Changes to Manuscript>Page 25>Discussion>Lines 16-25: Lifestyle related information was very limited and restricted to such as smoking status and alcohol consumption, which was available only in three study populations which limited risk factor comparisons. Detailed data on quantity, duration and pattern of tobacco and alcohol consumption are needed to understand better the observed associations, notably the observed protective effect of current smoking. Importantly, information on other lifestyle factors (such as dietary intake, air pollution and physical activity levels) were missing, which are likely to have significant impact on multimorbidity prevalence (47). Previous evidence suggests that effects of urbanisation on NCD prevalence is increasing rapidly in southern sub-Saharan Africa, we were unable to explore such regional differences with the limited data available(48).

**Reviewer #3**

Thank you for the opportunity to review the manuscript entitled, “Epidemiology of Multimorbidity in low-income countries of sub-Saharan Africa: findings from four population cohorts.” The authors present interesting data around common morbidities across a series of cohorts in 4 countries and provide age-standardized prevalence; the graphic (figure 1) is very useful and clear. Further, they present a series of potential risk and demographic factors. Interestingly, some of the expected relationships were inverse in some populations (e.g. higher education associated with higher prevalence of morbidity). There are a few minor areas for consideration and clarification.

MAJOR:

• In alignment with PLoS Global Public Health journal requirements, the authors need to clarify the availability of data. The current statement says, “The data that support the findings of this study are available from respective data controllers subject to successful registration and data governance application process.” The authors need to specify where data can be found and who controls the access process. This should be added in the data availability statement with weblinks/webpages or the process for requesting data.

**Author response**: Thank you for your useful suggestions. The data availability statement has been updated with further information and weblinks.

Data availability statement: The data that support the findings of this study are available from respective data controllers subject to successful registration and data governance application process.

1. For data access to Kiang West Longitudinal Population Study (The Gambia), please contact MRC unit The Gambia <https://www.lshtm.ac.uk/research/units/mrc-gambia/research-platforms-and-clinical-cohorts> .

2. For data access to Malawi Epidemiology and Intervention Research rural and urban cohorts, please contact <https://datacompass.lshtm.ac.uk/id/eprint/961/> .

3. For data access to General Population Cohort (Uganda), please contact <https://www.lshtm.ac.uk/research/centres-projects-groups/general-population-cohort> .

• In Table 4, the authors calculate the multimorbidity prevalence (any combination of 2 or more LTC) by country. However, each country had a different number of underlying morbidities assessed. As part of the limitations, can the authors provide any potential risk or limitation of comparing these prevalence estimates across countries? In Malawi there are more morbidities assessed and therefore, higher likelihood for these participants to be classified as having met the multimorbidity definition. If these authors do not think this is a risk or potential source of bias, please provide further detail.

**Author response**: We agree with the authors, we have acknowledged this in the limitations now.

Changes to Manuscript>Page25>Lines 14-17>Discussion: There were some differences in the underlying long-term conditions assessed across different countries which is likely to have impacted the overall prevalence of multimorbidity in each of the cohort. This introduces a possible source of bias when multimorbidity prevalence is controlled across different cohorts.

MINOR:

• In the Introduction, pleases clarify which “Academy of Medical Sciences” is referenced.

**Author response**: This has been amended.

Changes to Manuscript>Page4>Lines 1-2>Introduction: Multimorbidity, the presence of >2 long-term conditions (LTCs), has been identified as a current and increasing global health challenge by the United Kingdom Academy of Medical Sciences (1).

• Please consider adding the country names in the first paragraph of the ”Materials and Methods” section for consistency and specificity.

**Author response**: This has been corrected as stated above, thanks.

Changes to manuscript>Page6>Lines 1-4: We used cross-sectional data from: the Malawi Epidemiology and Intervention Research rural and urban cohorts, 2013-2017 (https://datacompass.lshtm.ac.uk/961/); the Kiang West Longitudinal Population Study (The Gambia); and the General Population Cohort in Kyamulibwa (Uganda).

• It is a bold statement to say “this is the first three country comparative study” when there are several systematic reviews noted and data from other African countries highlighted in the discussion. Consider rephrasing or be clear about where searches were conducted to state that this is the first study.

**Author response**: We have rephrased this sentence and we no longer claim this, thanks.

Changes to manuscript>Page25>Lines 2-6> Discussion: We described and compared, ~~This is the first study to our knowledge describing and comparing~~, with appropriate age-standardization, the epidemiology of multimorbidity in sub-Saharan Africa in three different countries including data from four populations (one urban and three rural) and representation from a wide spectrum of age groups, men and women and including detailed self-reported and measured multimorbidity outcomes.

• In the strengths and limitations section, it would be useful it the authors noted directionality of the impact on estimates by the differences in protocol for measurement of biomarker and anthropometric assessment.

**Author response**: This has been now acknowledged as a limitation.

Changes to manuscript>Page25>Lines 19-20> Discussion: There were some differences in protocols for measurement of anthropometric and biomarker measurements, which in turn could impact directionality and prevalence estimates (and observed variations) between study populations.
